# Detecting microstructural deviations in individuals with deep diffusion MRI tractometry

**DOI:** 10.1038/s43588-021-00126-8

**Published:** 2021-09-22

**Authors:** Maxime Chamberland, Sila Genc, Chantal M. W. Tax, Dmitri Shastin, Kristin Koller, Erika P. Raven, Adam Cunningham, Joanne Doherty, Marianne B. M. van den Bree, Greg D. Parker, Khalid Hamandi, William P. Gray, Derek K. Jones

**Affiliations:** 1 Cardiff University Brain Research Imaging Centre (CUBRIC), School of Psychology, Cardiff University, Cardiff, UK; 2 Donders Institute for Brain, Cognition and Behavior, Radboud University, Nijmegen, the Netherlands; 3 Image Sciences Institute, University Medical Center Utrecht, Utrecht, the Netherlands; 4 Department of Neuroscience, University Hospital of Wales (UHW), Cardiff, UK; 5 Bernard and Irene Schwartz Center for Biomedical Imaging, Department of Radiology, New York, NY, USA; 6 Medical Research Council Centre for Neuropsychiatric Genetics and Genomics, Division of Psychological Medicine and Clinical Neurosciences, Cardiff University, Cardiff, UK; 7 Brain Repair and Intracranial Neurotherapeutics (BRAIN) Unit, School of Medicine, Cardiff University, Cardiff, UK

## Abstract

Most diffusion magnetic resonance imaging studies of disease rely on statistical comparisons between large groups of patients and healthy participants to infer altered tissue states in the brain; however, clinical heterogeneity can greatly challenge their discriminative power. There is currently an unmet need to move away from the current approach of group-wise comparisons to methods with the sensitivity to detect altered tissue states at the individual level. This would ultimately enable the early detection and interpretation of microstructural abnormalities in individual patients, an important step towards personalized medicine in translational imaging. To this end, Detect was developed to advance diffusion magnetic resonance imaging tractometry towards single-patient analysis. By operating on the manifold of white-matter pathways and learning normative microstructural features, our framework captures idiosyncrasies in patterns along white-matter pathways. Our approach paves the way from traditional group-based comparisons to true personalized radiology, taking microstructural imaging from the bench to the bedside.

Normative modeling is an emerging statistical framework that aims to capture variability by comparing individuals with a normative population^1^. The benefits of normative modeling in neuroimaging in general are well documented, with applications in atypical brain development and psychiatry (see ref. ^2^ for a review). Applications of deep normative modeling have mostly been demonstrated at the voxel level using functional MRI data^3^ and volumetric data derived from standard structural magnetic resonance imaging (MRI)^4,5^.

Diffusion MRI (dMRI) is another MRI modality that allows non-invasive characterization of tissue microstructure. In the brain, for example, information about the structural architecture of the white matter can be obtained by probing the random motion of water molecules^6^ and acquiring multiple magnetic resonance images with different diffusion-senitization properties. The ability to derive semiquantitative features such as fractional anisotropy^7^ or mean diffusivity^8^, or to virtually reconstruct white-matter pathways with tractography^9^ has had a huge impact on the ability to distinguish between typical and atypical brain structures in vivo in health and disease^10^; however, prediction modeling (or case-control differentiation), in which a group of *N* patients with the same disease is compared with a group of *N*-matched controls, is not well suited to clinically heterogeneous groups (for example, neurological disorders^11^, psychiatric disorders such as schizophrenia or autism spectrum disorder, and rare cases). Despite decades of progress in the research domain, the primary clinical use of dMRI has been for largely limited to diagnosing acute ischemic stroke or grading and monitoring of tumor invasion. Several studies have shown success in identifying subtle but important microstructural changes at the individual patient level^12^, showcasing the potential of dMRI to be applied more broadly across applications. Yet there is a scarcity of dMRI frameworks for single-patient analysis (that is, one patient versus *N* controls). There is an urgent yet unmet need for a paradigm shift from group-wise comparisons to individualized diagnosis, that is, detecting whether (and where) the tissue microstructure of a single participant is abnormal^13^; not only would this greatly facilitate the study of clinically heterogeneous groups^1,4^, it would also facilitate the study of rare diseases and true clinical adoption (that is, making a diagnosis/prognosis in an individual patient).

As mentioned above, current efforts to apply deep normative modeling to neuroimaging have so far relied on voxel-based methods. For the assessment of white matter—which comprises continuous pathways—under conditions in which an entire pathway may be affected (for example, in developmental disorders such as schizophrenia^14^), fragmenting the analysis in this way is suboptimal, whereas operating on the manifold of reconstructed tracts offers a more intuitive approach. Indeed, in comparing microstructural properties between groups, many computational pipelines adopt a tractometry approach^15,16^, that is, mapping measures along pathways reconstructed via tractography, either by averaging along the whole tract^17^ or a segment thereof^18–21^. Along-tract profiling has been applied previously to investigate various brain conditions^15,19,22–24^. The main advantage here is that image registration can be avoided as tractometry is performed in each patient’s native space, resulting in a set of individual tract profiles. There are, however, some limitations. First, most analyses treat tractometry measures from specific pathways as independent measures. This univariate approach has the potential to obscure key relationships between different tracts.

Focusing on any particular anatomical location therefore increases the risk of losing the full picture. Although individual pathways can appear normal in isolation, by considering them as an ensemble (for example, as part of a network), any such intertract relationships could collectively help to identify outliers. Second, when analyzing multiple measures (even when derived within the same tract), statistical analysis is hampered by: (1) the multiple comparisons problem; and (2) any covariance between measurements^17,25^. Here multidimensional approaches can increase statistical power by combining the sensitivity profiles of independent modalities^25–27^.

Here we present an anomaly detection framework (Detect) that uses a data-driven, unsupervised normative modeling approach based on deep autoencoders^28^. Autoencoders are a type of artificial neural network traditionally used for dimensionality reduction^28^. They can capture non-linear interactions between the input features by learning a self-representation of their inputs through a low-dimensional layer (Fig. 1); the goal is to generate an output $\left(\hat{x}\right)$ similar to the input (**x**) by minimizing the reconstruction error. This same representation can be exploited for anomaly detection by analyzing deviations in the reconstruction. Detect moves substantially beyond dMRI group-level analysis techniques by identifying and localizing anomalies in multiple tract profiles at once at the individual level. The proposed framework was trained to learn normative sets of features derived from healthy brain tract profiles in three independent datasets and one reproducibility dataset^29^. Once trained, the network is then presented with unseen healthy tract profiles (for testing) and subsequently exposed to tract profiles from individuals diagnosed with neurological/psychiatric conditions. We emphasize that the model has no access to the diagnostic labels during the training phase and thus our anomaly detection approach is fully unsupervised. To assess generalization, the framework was then applied to single participants with a range of neurological and psychiatric disorders, including children and adolescents with copy number variants (CNVs) at high risk of neurodevelopmental and psychiatric disorders; patients with drug refractory epilepsy; and patients with schizophrenia (SCHZ). We compared the performance of our approach with (1) a conventional *z*-score-distribution approach and (2) principal component analysis (PCA) combined with the Mahalanobis distance (a widely used approach in cluster analysis and classification techniques)^21,24,27^. Importantly, the analyses of the three pathological cases were performed in the browser, from the point-of-view of future Detect users.

## Results

### Framework overview

Detect is an open-source, user-facing tool built on the interactive Streamlit framework, which promotes data exploration through interactive visualizations in the browser. The framework offers three main scripts: Detect, Inspect and Relate. Detect facilitates patient comparisons using cross-validated area under the curve (AUC) computed over a set of user-defined iterations, by means of *z*-score, PCA or autoencoder (see Supplementary Fig. 4). The output is a single, bootstrapped anomaly score for each patient. Inspect allows the user to select a single subject and to compare it with the rest of the population. Here, feature anomalies are highlighted using a leave-one-out cross-validation approach, and only segments along the tract where two consecutive outliers occurred are reported. Results are displayed in real-time during each iteration for the user to evaluate. Furthermore, both scripts allow the visualization of tract profiles. Finally, Relate is a simple visual interface for correlating the anomaly scores obtained by the previous commands with clinical scores. In all scenarios, micro-structural features included fractional anisotropy, mean diffusivity and rotationally invariant spherical harmonic features of zeroth and second orders^30^ (RISH0 and RISH2, respectively) derived from the largest *b*-value data in each dataset to maximize sensitivity to the intra-axonal signal^31^.

### White-matter anomaly detection in CNV participants

#### Discriminating power

**Fig. 2:**
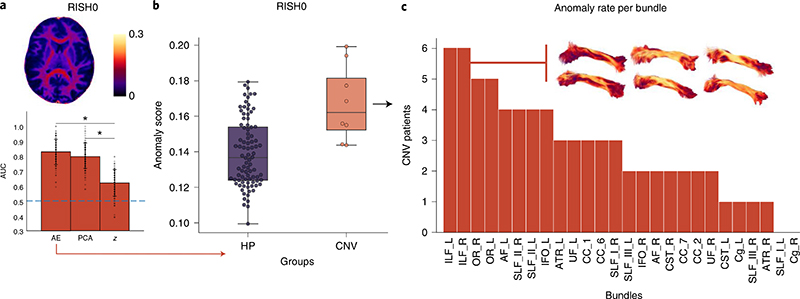
Anomaly scores for the CNV dataset. **a**, The autoencoder (AE) network and PCA approaches provided better discriminating power in terms of sensitivity/specificity tradeoffs compared with traditional linear univariate approaches (*P=5.69×10^-40^ and *P*=1.92×10^-33^, Bonferroni corrected with *α*=0.01, two-tailed *f*-tests for AE*-z* and PCA-*z*, respectively). The average AUC over 100 iterations (bottom) for the RISH0 feature (top) is displayed (data are presented as mean values ± s.d.). **b**, The RISH0 features show higher reconstruction error for the CNV (orange box plot) compared with the typically developing patients (purple box plot) with a precision-recall AUC of 0.45 (center line, median; box limits, upper and lower quartiles; whiskers, 1.5 interquartile range; *n* = 90 healthy participants and *n* = 8 CNVs). In comparison, a random classifier would score 0.08. The box shows the quartiles of the dataset whereas the whiskers extend to show the rest of the distribution. **c**, From a group perspective, anomaly rates were mostly observed in the ILF (color map: RISH0, lateral view), optic radiations and SLF. OR, optic radiations; UF, uncinate fasciculus; AF, arcuate fasciculus; IFO, inferior fronto-occipital fasciculus; CC, corpus callosum; Cg, cingulum; ATR, anterior thalamic radiation, R, right brain hemisphere; L, left brain hemisphere.

First we investigate individual differences in white-matter microstructure in children with CNVs at high genetic risk of neurodevelopmental and psychiatric disorders^32^, which are relatively rare and therefore challenging to recruit for research imaging studies^33^. Note that the framework was trained using data from typically developing children only. In general, the autoencoder approach showed higher AUC scores across the microstructural metrics and was better at identifying CNV patients as outliers, providing substantially higher sensitivity–specificity tradeoffs than the *z*-score and Mahalanobis-based approaches (Supplementary Fig. 1 and Supplementary Table 2). For example, the RISH0 feature showed higher discriminating power (AUC = 0.83 ± 0.08) compared with the mean univariate *z*-score (AUC = 0.80 ± 0.09) and multivariate Mahalanobis distance (AUC = 0.61 ± 0.09). In comparing the RISH0 group distributions (Fig. 2), anomaly scores derived via the autoencoder were significantly different (Kolmogorov–Smirnov test = 0.62, *P*< 0.003; Cohen’s *d* effect size = 1.39) between the CNV individuals and the typically developing patients. In particular, all CNV patients had an anomaly score larger than the typically developing patient mean and 50% of them were larger than the 95th percentile of the typically developing patient population. In comparison, the difference between the anomaly scores was less pronounced with the *z*-score (Kolmogorov–Smirnov = 0.34, *P* = 0.3, Cohen’s *d =* 0.38) approaches, whereas similar results were obtained by using the Mahalanobis distance (Kolmogorov–Smirnov = 0.56, *P =* 0.01, Cohen’s *d =* 1.20).

#### Tract-specific deviations

**Fig. 3:**
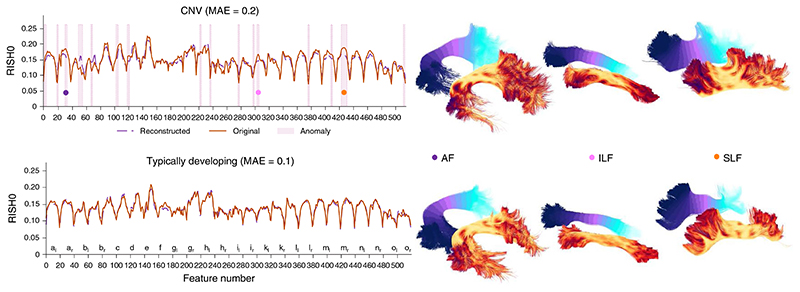
Along-tract anomalies in a single patient. Reconstructed tract profiles of CNV (top) and typically developing (bottom) patients reveals RISH0 discrepancies along various association bundles such as the AF, ILF and SLF. The marked pink areas represent sections along the bundle where anomalies are detected by the leave-one-out cross-validation approach (threshold = 1 per number of healthy participants). Specifically, the autoencoder is trained using healthy participant data only, and compared with the FCD patient. Then for each of the healthy participants, the FCD patient is shuffled back in the population and the newly left out healthy participant is compared with that population. We then assess the MAE. The letters correspond to the tract list found in the Supplementary Information.

A key advantage of using deep autoencoders for anomaly detection over traditional PCA-derived approach is its unique ability to relate the anomaly back to the individual elements of the input data. More specifically, the predicted data retains the same dimensionality as the input data and thus it is possible to see which feature cannot be accurately recovered by the autoencoder. For example, if a feature has a positive reconstruction error, then one can infer that the network learned a smaller value for that feature than what was provided as input. In the context of the CNV participants, multiple regions were highlighted (by positive reconstruction errors) as deviants from the typically developing patient population. Figure 3 reveals a high anomaly rate for various association bundles such as the bilateral inferior longitudinal fasciculus (ILF), optic radiations and the left superior longitudinal fasciculus (SLF_II). This is in line with current literature where microstructural differences are expected along association pathways, in agreement with psychotic symptoms^34^.

### White-matter anomaly detection in epilepsy

Focal cortical dysplasia (FCD)—a malformation of cortical development—is the most common etiology in drug-resistant neocortical partial epilepsies^35^. Although complete resection is the main predictor of seizure freedom following surgery, a substantial proportion of FCDs may be missed with standard clinical imaging protocols^35^ and the seizure-generating network may extend far beyond the visible dysplasia. Diffusion MRI contrast enhances the sensitivity of MRI to differences in the brain, but has only been reported at the group level^36^. Here we demonstrate two key advantages of the deep autoencoder approach in a clinical context. First, it succeeded in detecting white-matter anomalies that a conventional *z*-score-based approach has missed, potentially due to hidden interactions between the features. Second, detection of abnormal microstructural features away from putative seizure onset zone, as demonstrated in the first example, may contribute to the mapping of epileptogenic networks in individuals; thus, although the examples shown here had radiological changes detectable with T2-weighted sequences, the method could potentially be extended to cases of MRI-negative partial epilepsy increasing the diagnostic yield.

**Fig. 4:**
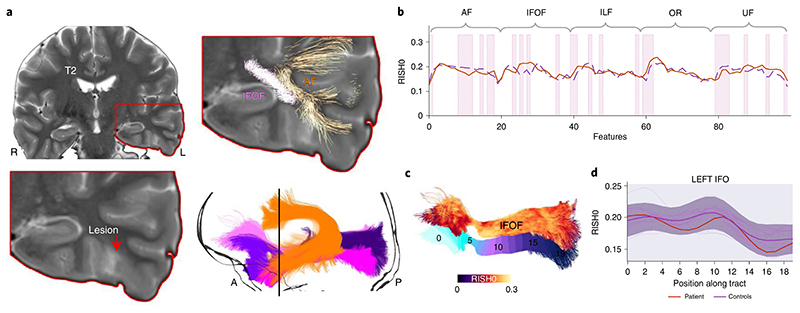
Focal cortical dysplasia anomaly detection (patient 1). **a**, The T2 hyperintense lesion, located at the base of the skull in the temporal lobe, is shown. **b**–**d**, Several pathways with anomalies interdigitate in the vicinity of the lesion. Although the inferior fronto-occipital fasciculus (**c**; IFOF with RISH0 colormap overlayed and the 20 along-tract sections underlayed) signal did not extend beyond the shaded areas (**d**; ±1*z*-score), the proposed anomaly detection framework identified abnormalities in that region (**b**, pink shaded areas; bold orange line, original tract-profiles; dotted purple line, reconstructed representation learnt from the network). R, right side; L, left side; A, anterior; P, posterior.

Patient 1 is a young adult female with seizures described as a fuzzy painful sensation in the torso rising up to the head, associated with mumbling sounds, occurring 2–5 times per day. A scalp video electroencephalogram (EEG) showed left temporal interictal epileptiform discharges and left temporal EEG onset. Clinical imaging demonstrated a small area of cortical–white-matter junction blurring in the laterobasal left temporal lobe associated with a transmantle area of T2 hyperintensity, suggestive of FCD type II^37^. Neuropsychological assessment was concordant with a left temporal deficit, also revealing preserved mesial structures manifesting in relatively preserved verbal memory performance. Subsequent stereo-EEG (SEEG) implantation confirmed ictal onset and prominent interictal discharges from neocortical contacts immediately behind the MRI lesion; furthermore, neocortical discharges were seen in SEEG contacts close to the temporal pole. Based on those clinical findings (neuropsychological assessment and SEEG), the patient proceeded to have resection with histology consistent with FCD. Five tracts of possible relevance were interrogated (Fig. 4). Microstructural anomalies were identified along the left ILF and optic radiations in the immediate proximity of the T2-weighted changes corresponding to SEEG contacts with maximal ictal EEG changes. Anomalies in the temporal portions of the left inferior fronto-occipital fasciculus and uncinate fasciculus pointed towards the temporal pole corroborating the SEEG findings that, despite normal clinical MRI, this area was part of the seizure network.

**Fig. 5:**
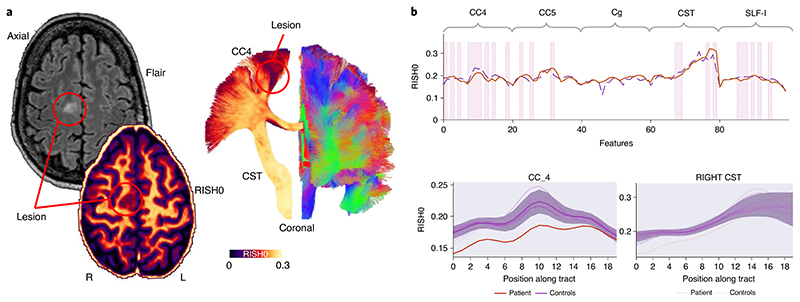
FCD anomaly detection (patient 2). **a**, The lesion is located anterior to the right primary motor cortex in the supplementary motor area (hyperintense signal on the FLAIR image, hypointense on the RISH map). Tractography show tracts traversing the area (CC4). **b**, Anomalies were identified in the right CC4, CST and SLF-I bundles (top, pink shaded areas). The bold orange line represents the original tract-profiles whereas the dotted purple line represents the reconstructed representation learned from the network. The *z*-score approach shows less focused anomaly patterns along the tracts (shaded area = ±1*z*-score).

Patient 2 is an adult female with focal onset seizures since childhood occurring daily with episodes of loss of contact, grimacing and limb stiffening hypermotor movements, including clutching at nearby object on the left side. Scalp video EEG findings were consistent with frontal onset seizure semiology. Clinical MRI showed blurring of the cortical–white-matter junction between the right posterior superior frontal gyrus and the adjacent precentral gyrus, and a transmantle sign on T2/FLAIR from the cortex reaching all the way to the lateral ventricle, consistent with FCD type II. Subsequent SEEG recordings demonstrated spatial overlap between primary motor areas and early ictal onset and hence the patient did not proceed to surgery. Five tracts of possible relevance were interrogated with our framework (Fig. 5). Anomalies were detected corresponding to radiological and electrophysiological findings along the right corticospinal tract (CST), primary motor (CC4) and superior longitudinal fasciculus (SLF-I) beyond the visible lesion. No anomalies were found along the right cingulum and primary sensorimotor (CC5) regions.

The results are promising, with the tool identifying anomalies in concordance with clinical hypothesis in a single-patient analysis paradigm, testifying to its utility for clinical evaluation. Its extra value is highlighted by its sensitivity to outlying tract segments not detected with the conventional *z*-score approach. The *N =* 1 approach to detect deep white-matter anomalies illustrated here will facilitate the identification of individualized therapy most appropriate to that patient, suggesting additional targets for diagnostic evaluation and possible surgical treatment.

### Linking brain heterogeneity with epidemiological findings in schizophrenia

**Fig. 6:**
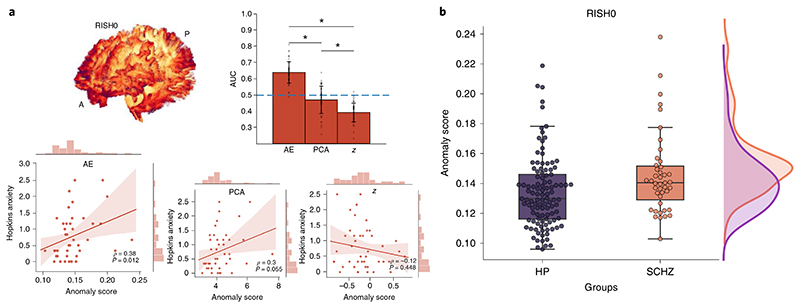
Anomaly scores for the SCHZ cohort. **a**, The autoencoder provides a better discriminating power compared with traditional linear univariate and multivariate approaches with a mean AUC of 0.64 ± 0.06 (bar graph, *P=1.00×10^-33^, *P*=1.05×10^-5^ and *P*=1.63×10^-26^, Bonferroni corrected at *α* = 0.01, two-tailed *t*-tests for AE-PCA, AE-*z* and PCA-*z*, respectively). For illustrative purposes, anomaly scores derived from the autoencoder, PCA and z-score are correlated with the Hopkins anxiety score (shaded area: 95% confidence interval). **b**, The RISH0 features show higher reconstruction error (100 averages) for the SCHZ than the healthy participants (*t* = -2.48, *P* = 0.01, Cohen’s *d* = 0.47, two-sided *t*-test; center line, median; box limits, upper and lower quartiles; whiskers, 1.5×interquartile range; *n* = 109 healthy participants and *n* = 43 SCZH, respectively).

The extent to which individual clinical variability in schizophrenia relates to microstructural variability remains a key challenge in neuropsychiatry^38^, with most findings being at the group or voxel level. For the RISH0 feature, the autoencoder approach was better at identifying single SCHZ patients as outliers (AUC = 0.64 ± 0.06) when compared with PCA (AUC = 0.59 ± 0.07) or the *z*-score (AUC = 0.39 ± 0.06). In comparing these group distributions, anomaly scores derived from the autoencoder were found to be significantly different *(t*= −2.60, *P =* 0.01, Cohen’s *d =* 0.47) between the SCHZ individuals and the healthy participants (Fig. 6). In particular, 31 of the 43 SCHZ patients had an anomaly score larger than the healthy participants’s mean and nine of them were larger than the 95th percentile of the healthy participants’s population. In comparison, the difference between the anomaly scores was less pronounced for the PCA (*t*= −1.75, *P =* 0.08, Cohen’s *d =* 0.32) and *z*-score (*t* = 1.85, *P =* 0.07, Cohen’s *d =* 0.33) approaches (see Supplementary Fig. 1 and Supplementary Table 2). Furthermore, the above chance-level detection rates of the proposed deep autoencoder in SCHZ suggest a successful application of the tractometry-based framework in unsupervised anomaly detection. The significance of these results are even more pronounced considering the challenging task at hand; that is, where even a supervised support vector machine classifier provides similar accuracy (AUC = 0.65 ± 0.13). Finally, to provide an illustrative way in which the Detect tool can provide an important avenue for future studies, anomaly scores were correlated with clinical scores. The Hopkins anxiety index (Hopkins symptom checklist), a widely used screening instrument to study mental illness, was used as a proof of concept. For the autoencoder, PCA and *z*-score, the Spearman’s rank correlation coefficients were *ρ* = 0.38 (*P* = 0.012), *ρ* = 0.3 (*P* = 0.055) and *ρ* = −0.12 (*P* = 0.448), respectively (Fig. 6).

### Repeatability of anomaly scores and tract profiles

Using a test-retest dataset (six patients, five time points^29^), we assessed the repeatability of (1) the input RISH0 tract profiles and (2) the generated anomaly scores by calculating the intraclass correlation coefficient (ICC, with two-way mixed, absolute agreement for average measurements as in ref. ^29^) and the coefficient of variation (CoV). Supplementary Fig. 2 shows the repeatability of the tract profiles, with the optic radiations being the most reproducible bundles (mean ICC = 0.95, CoV = 0.03) and the left cingulum being the least reproducible (ICC = 0.66, CoV = 0.06). In terms of anomaly scores, the proposed anomaly detection framework shows reconstruction errors that are reproducible across sessions (Supplementary Fig. 3) with an ICC of 0.96 (95% CI: 0.88, 0.99) and a CoV of 0.06. Furthermore, reliability of anomaly scores in the pediatric dataset can be estimated from the test-retest dataset. Using the two-way mixed ICC for consistency (for single measurements) of 0.85 from the test-retest study, assuming a similar measurement-related variance and accounting for the standard deviations in both cohorts, the ICC for anomaly scores in the pediatric cohort is 0.86. Even if the measurement-related variance was to increase by 30%, the ICC would still indicate good reliability (0.76). This confirms that the demonstrated group differences in anomaly scores are unlikely to have occurred due to measurement-related variance.

## Discussion

Further exploration of input features and hyperparameters of the model remains to assess the generalizability of the framework and its application to other pathologies. From a generalization point of view, the key problem in biomarker research is the need for individual prediction/diagnosis. Advancing knowledge of brain pathology and related cognitive impairment at the individual level is essential for early detection and intervention. Normative models show that, if groups are too heterogeneous, it can be a challenging task to learn characteristics from a given population using supervised approaches, hence the need for unsupervised learning^1^. Although the amount of data we can employ in imaging studies is relatively small in comparison with population-based studies, the framework provides a principled method to detect individual differences in tissue microstructure. With the ever-growing amount of dMRI data being acquired, the framework will make less conservative inferences as the number of data points increases. In principle, our framework can be trivially extended to tract-based assessment of non-diffusion-based microstructural metrics (for example, such as magnetization transfer and myelin water imaging^16,17^), as long as the associated quantitative maps are co-registered with the diffusion space. They could be derived from any manually defined or atlas-derived regions of interest, tract-based regions of interest as done here, or even at the voxel-based level^39^. Moreover, our framework could be applied to any set of neuroimaging features. For example, these inputs could include time series from functional MRI and cortical thickness derived from structural T1 imaging. Finally, other applications of the framework include the characterization of microstructural changes in neurological disorders without gross pathology. Furthermore, Detect could also help predict worse phenotypic outcome in those with a rare genetic disorder (for example, CNV carriers) and potentially allow for early therapeutic planning in the future.

One of the limitations of single-patient analysis is that it requires substantial amounts of healthy participant data to define a normative brain^1^. Combining multisite or multiscanner dMRI datasets can greatly increase the statistical power of neuroimaging studies^40^, but cross-scanner and cross-protocol variability challenges joint analysis, hence the need for data harmonization^30,41^. However, dMRI harmonization is a young field, there is not yet an established method that can bring cross-scanner variability back to the level of within-patient variability. Moreover, tractography still faces considerable challenges in the field^42,43^ and most commonly available tools can only track reliably within normal-appearing white matter. Although recent machine learning approaches have shown promise in reproducible tract segmentation across patients^44^, therefore strengthening hope of analyzing dMRI data for group studies, there is a potential challenge: along-tract profiling approaches should only be used when a complete tract has been reconstructed. Future work will establish the utility of this approach in conditions where pathology leads to incomplete tract reconstruction. Another limitation is that autoencoders require more computation than PCA; however, PCA will also have limitations for large datasets where memory storage is an issue.

The choice of dMRI measure will also influence the capacity of the tool to detect anomalies. For example, the use of fiber-specific measurements^45,46^ could help better disentangle anomalies in fiber-crossing regions. This would of course demand a model-based approach as opposed to the RISH0 features employed in this study. Furthermore, it was recently demonstrated that there is more sensitivity to individual differences at high *b*-values^31,47^. Nevertheless, we are encouraged to see that even with more commonly used *b*-values (for example, *b* = 1,000 s mm^–2^), we are still able to uncover patient/ control differences in the SCHZ cohort, highlighting the potential for widespread clinical adoption of dMRI.

In the context of microstructural MRI, the demonstration of Detect through the three different scenarios goes beyond the utility of other techniques and provides compelling motivation for future application. The unsupervised multivariate framework proposed here uses state-of-the-art machine learning to approach high-dimensional data non-linearly and improve accuracy and precision over traditional anomaly detection. Our deep learning approach also provides advantages over other statistical approaches for outlier detection as it was recently shown (using functional MRI data^3^) that deep unsupervised approaches improve identification of psychiatric patients compared to mass-univariate normative modeling. It is also generally accepted that autoencoders tend to perform better when the middle layer (that is, bottleneck layer) is small when compared with PCA. This can potentially mean that the same accuracy can be achieved with less components and hence may be beneficial for smaller datasets.

Browser-based applications are becoming increasingly popular among the computational neuroscience community due to their ease of use and accessibility across devices^21^. Detect enables the detection of abnormalities in clinically heterogeneous groups or rare cases and ultimately improve diagnosis of neurological and neuropsychiatric disorders. Our aim was to develop and distribute an open-source framework to characterize microstructural white-matter changes at the individual level. This enables the detection of abnormalities To the best of our knowledge, other tools such as AFQ Browser21 compare individuals using a linear approach (*z*-score) that considers each tract-segment independently and ignores potential complex interactions between the features. Those tract segments are then statistically tested in an univariate manner, and as such the correction for multiple comparisons—required by the typical high dimensionality of dMRI data—will hamper the discriminating power of the analysis^25^. Recently, PCA was employed to acknowledge the multivariate nature of dMRI data^25–27^, but this approach still relies on linear assumptions thereby ignoring possible complex interactions between the features. We believe strongly that the proposed deep autoencoder approach goes hand-in-hand with existing browser-based dMRI analysis frameworks21 in encouraging reproducible research and data-driven discoveries. Finally, we encourage future users of Detect to apply the tool to their own datasets and we welcome contribution to the tool in the form of added functionalities via GitHub.

In summary, diffusion MRI offers great promise to detect subtle differences in tissue microstructure when applied at the group level; however, the goal of clinical neuroimaging is to be applicable at the individual level. The single case approach proposed here will facilitate the identification of individualized therapy most appropriate to that patient, forming a baseline biomarker for subsequent monitoring through a therapeutic process. We believe that our tractometry-based anomaly detection framework paves the way to progress from the traditional paradigm of group-based comparison of patients against healthy participants, to a personalized medicine approach, and takes us a step closer in transitioning microstructural MRI from the bench to the beside.

## Methods

### Detect interactive interface

Users of Detect will input demographic data that consist of comma-separated values (.csv), where each row represents a patient (ID). Example demographics columns include: group, age, gender or clinical scores. The user is given the option to correct for confounding factors (that is, by treating those attributes as covariates across the entire brain^2^), resulting in age-independent microstructural features, for example. The microstructural tractometry data format consists of an .xlsx spreadsheet, where each sheet represents a dMRI metric (for example, fractional anisotropy, mean diffusivity and so on). As per the demographic data, patients are stacked individually on each row. The first column denotes the ID of each patient. The remaining columns follow the following convention: bundle_hemi_section where bundle is the white-matter bundle of interest, hemi is the hemisphere (that is, left or right and void for commissural tracts), and section is the along-tract portion (for example, from 1 to 20).

### Data acquisition and preprocessing

*CNV dataset*. Diffusion MRI data were acquired from 90 typically developing children (age 8–18 years) and eight children with CNVs at high risk of neurodevelopmental and psychiatric disorders (CNVs, 2× 15q13.3 deletion, 2× 16p11.2 deletion, 3× 22q.11.2 deletion and 1× Prader-Willi syndrome) and no apparent white-matter lesions (age 8–15 years). Data collection procedures for the typically developing and CNV groups were approved by the Cardiff University School of Psychology and School of Medicine Ethics Committees, respectively. Children under 16 and those over 16 who lacked capacity to consent given written/verbal assent and their parents or legal guardians gave written consent on their behalf. Images were acquired using a Siemens 3 T Connectom MRI scanner (32-channel radiofrequency coil, Nova Medical) with 14 b0 images, 30 directions at *b* = 500, 1,200 s mm^−2^; 60 directions at *b* = 2,400, 4,000, 6,000 s mm^−2^; and 2 × 2 × 2 mm^3^ voxels (TE (echo time)/TR (repetition time) = 59/3,000 ms; Δ/*δ* = 24/7 ms). The total scan time for the multishell protocol was 16 min and 14 s. Each dataset was denoised^48^ and corrected for signal drift^49^, motion and distortion in FSL^50^, gradient non-linearities^51^ and Gibbs ringing^52^. Next, RISH features^30^ were derived for each patient using the *b* = 6,000 s mm^−2^ shell to maximize sensitivity to the intra-axonal signal^31^ (zeroth and second orders only, RISH0 and RISH2, respectively). Furthermore, diffusion tensors were generated using an in-house non-linear least squares fitting routine using only the *b* ≤ 1,200 s mm^−2^ data, followed by the derivation of fractional anisotropy and mean diffusivity maps.

#### Epilepsy dataset

Diffusion MRI data from two epilepsy patients with FCD were acquired on a Siemens 3 T Connectom MRI scanner with 60 directions at *b* = 1,200, 3,000 and 5,000 s mm^−2^ and 1.2 × 1.2 × 1.2 mm^3^ voxels (TE/TR = 68/5,400 ms; Δ/*δ* = 31.1/8.5 ms; total scan time = 30 min and 25 s). Furthermore, data on 15 healthy participants (aged 21–41 years) from the computational diffusion MRI harmonization database were used^40^. Data collection procedures for the healthy participant and FCD groups were approved by the Cardiff University School of Psychology and School of Medicine Ethics Committees, respectively. Written informed consent was obtained from all patients. Each dataset was corrected for Gibbs ringing^52^, signal drift^49^, motion and distortion in FSL^50^, and gradient non-linearities^51^. Next, RISH0 features^30^ were derived for each patient using the *b* = 5,000 s mm^−2^ shell. A fourfold data augmentation was applied to the healthy participant tract profiles using the synthetic minority oversampling technique^53^ resulting in 75 healthy participants.

#### SCHZ dataset

Diffusion MRI data from the UCLA Consortium for Neuropsychiatric Phenomics^54^ was downloaded from the OpenNeuro platform (openneuro.org/datasets/ds000030/versions/00016), which also contains demographic, behavioral and clinical data. Although more focused on functional MRI, the dataset contains dMRI data from 123 healthy participants and 49 individuals with SCHZ amongst other psychiatric disorders. Data were acquired on a Siemens 3 T Tim Trio MRI scanner with one b0 image, 64 directions at *b* = 1,000 s mm^−2^ and 2 × 2 × 2 mm^3^ voxels. Data quality assessment was first performed, resulting in the exclusion of datasets with reduced field-of-view (preventing the reconstruction of white-matter bundles in the inferior temporal lobes) and those with substantial slice dropout (impacting estimation of diffusion metrics). A total number of 109 healthy participants (aged 21–50 years) and 43 SCHZ patients (aged 22–49 years) were used for further analysis. Diffusion data were denoised^48^, corrected for patient motion in FSL^50^ and distortion using the anatomical T1-weighted image as reference. Next, RISH features (RISH0, RISH2) were derived for each patient. Finally, diffusion tensors were generated using iteratively weighted least squares in MRtrix^55^ followed by the derivation of fractional anisotropy and mean diffusivity maps.

#### Repeatability dataset

To assess repeatability, we employed the microstructural image compilation with repeated acquisitions dataset^29^, which comprises five repeated sets of microstructural imaging in six healthy human participants (three female, aged 24–30 years). Each participant was scanned five times in the span of two weeks on a 3 T Siemens Connectom system with ultra-strong (300 mT m^-1^) gradients. Multishell dMRI data were collected (TE/TR = 59/3,000 ms; voxel size = 2 × 2 × 2 mm^3^; *b*-values = 0 (14 volumes), 200 and 500 (20 directions), 1,200 (30 directions), 2,400, 4,000, and 6,000 (60 directions) s mm^−2^) resulting in a total scan time of 16 min and 37 s. The same preprocessing as the CNV dataset was applied. Data collection was approved by the Cardiff University School of Psychology Ethic Committee and written informed consent was obtained from all patients.

### Tractometry

For each dataset, automated white-matter tract segmentation was performed using TractSeg^44^ (see Supplementary Table 1) using multishell constrained spherical deconvolution^47^. For each bundle, 2,000 streamlines were generated. Tractometry^16^ was performed (sampling fractional anisotropy, mean diffusivity, and RISH0 and RISH2 at 20 locations along the tracts^19,23,^^25^) using a Nextflow architecture provided by SCILPY (github.com/scilus/scilpy). Specifically, individual streamlines were reordered for all patients to ensure consistency using the following order: left-to-right for commissural tracts, anterior-to-posterior for association pathways and top-to-bottom for projection pathways. Next, a core streamline was generated and microstructural metrics at each vertex of the bundle were projected to the closest point along the core. The resulting tract profiles were concatenated to form a feature vector (*x*).

### Artificial neural network

Our autoencoder implementation consists of a symmetric design of five fully connected layers (l_*n*_). The input and output layers (l_1_ and l_5_) have exactly the same number of nodes as the number of input tracts features. The inner layers (l_2_ and l_4_) consecutively apply a compression ratio of two by reducing the number of nodes by half, up to the bottleneck hidden layer (l_3_). For example, if using an input vector made of 100 features, l1 and l_5_ will consist of 100 nodes; l_2_ and l_4_ 50 nodes; and 25 nodes for the bottleneck l_3_. Rectified linear units activation was used between the layers to promote sparse activation and tan*h* for the last layer (epochs = 25, batch size = 24, learning rate = 1 × 10^-3^; optimizer, Adam; loss, mean squared error; validation split = 0.1). Using different activation functions in different layers aims at balancing the advantages and disadvantages of the two activation functions. To promote sparsity and reduce overfitting, an activation penalty was imposed to the bottleneck layer using *ℓ*_1_-regularization (×10^−5^). This is especially best suited for models that explicitly seek an efficient learned representation. The goal is to generate an output $\left(\hat{x}\right)$ similar to the input (*x*) by minimizing the reconstruction error. Here the MAE was used as anomaly score and is defined as:

(1) $$\text{MAE}=\frac{1}{n}\overset{n}{\underset{j=1}{\sum^{\text{​}}}}|x_{i}-{\hat{x}}_{i}|$$

The MAE measures the average magnitude of the errors and is derived during testing by computing the absolute differences between the reconstructed microstructural features $\left({\hat{x}}_{i}\right)$ and the raw input features (*x_i_*). Due to the heavily imbalanced group ratio between healthy participants and patients (that is, CNV and epilepsy), a bootstrap was implemented to draw random samples of equal sizes from each group. Specifically, the autoencoder is trained using healthy participants data only. The entire dataset is therefore first split into a training set (80%, made of healthy controls only) and a validation set (20%, by combining the patients with a matching number of healthy participants) to create the outer fold; 10% of the training set is held out for testing during the training phase (inner fold) to evaluate the loss. Age and sex regression^56^ and feature normalization (min–max) were performed on the normative training set and subsequently applied to the held-out validation set to prevent information leakage. To derive conservative estimates and assess variations within the model, we repeated this process 100 times and report the mean MAE for each patient. Finally, we compared the sensitivity versus specificity of the anomaly scores using the mean receiver operating characteristic AUC across iterations, with standard deviations used as uncertainties. Comparisons of the AUC scores between patients and controls were performed using two-tailed *t*-tests assuming equal variances in the case of balanced groups and Kolmogorov–Smirnov test for the unbalanced groups. The correlation between anomaly scores and clinical scores was computed using Spearman’s *ρ*.

### Univariate approach

One of the most commonly used tools in determining outliers is the *z*-score. The *z*-score (or standard score *z* = *x − μ*)/*σ*) is a way of describing a data point as deviance from distribution, in terms of standard deviations from the mean of the normal distribution. Here, *z*-scores were computed for each tract-segment, relative to the mean of the healthy group (*μ*) and averaged to derive a patient-specific anomaly score at each iteration (described above). The mean over all iterations described above was retained as anomaly scores for all patients.

### Multivariate linear approach

Principal component analysis was applied to the set of features by restricting the dimensionality to preserve features accounting for 85% of the variance in the data. In Detect, this number can be manually defined as the percentage of explained variance. Next, the Mahalanobis Distance (*M*, a multidimensional generalization of the z-score that accounts for the relationships between the white-matter bundles) was used to derive an anomaly score defined as:

(2) $$M(x)=\sqrt{{\left(x-\mu \right)}^{\prime \;{\;}^{.}}C^{-1\;}{}^{{}^{.}}(x-\mu )},$$

where *x* represents the feature vector of a given patient, *μ* is the vector of mean microstructural metrics for each tract location *s*, and *C*^−1^ is the inverse covariance matrix of the input features. The problem of anomaly detection can be seen as a one-class classification problem and therefore, our training data only contains healthy participants to calculate *C*. an *M* score was then derived for all unseen patients in relation to the healthy participant distribution. The same dataset split as aforementioned was used to derive a bootstrapped estimate of *M* for each patient, which was subsequently analyzed.

### Support vector machine comparison

A supervised support vector machine classifier was used for comparisons on the SCHZ dataset. Class weights were set to account for the class imbalance between healthy participants and patients. The classifier was validated using a repeated (ten times) stratified, fivefold crossvalidation approach in scikit-learn (scikit-learn.org). Optimized parameters were derived for each cross-validation fold using a grid-search approach. Those included the choice of kernel ([radial basis function, linear]), regularization ([1, 10, 100, 1000]) and gamma parameters ([10^−3^, 10^−2^, 10^−1^, 1, 10^1^, 10^2^, 10^3^]).

## Supplementary Material

The online version contains supplementary material available at https://doi.org/10.1038/s43588-021-00126-8.

146348_SD_Fig_2

146348_SD_Fig_3

146348_SD_Fig_4

146348_SD_Fig_5

146348_SD_Fig_6

146348_Sup_Material

## Figures and Tables

**Fig. 1:**
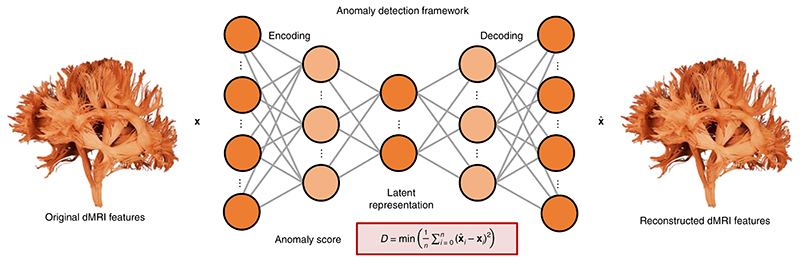
Graphical representation of the proposed anomaly detection framework. The neural network consists of a deep autoencoder symmetrically designed with five fully connected layers. The input and output layers have exactly the same number of nodes as the number of input tracts features (|**x**|, represented by the colored vector). The goal of the network is to generate an output $\left(\hat{x}\right)$ similar to the input (**x**) by minimizing the reconstruction error (*D*). Here, the mean absolute error (MAE) was used as the anomaly score; MAE measures the average magnitude of the errors and is derived during testing by computing the absolute differences between the reconstructed microstructural features $\left({\hat{x}}_{i}\right)$ and the raw input features (**x***_i_*,). The number of input features is denoted by *n*, with the current feature denoted by *í*. Node coloring is for illustrative purposes only.

## Data Availability

Original datasets are accessible through the original publications, including the MICRA^29^ repeatability dataset (osf.io/z3mkn/) and the CNP^54^ dataset (https://openneuro.org/datasets/ds000030/versions/1.0.00). The cDMRI dataset is publicly available and information on how to obtain the data can be found on the following webpage: https://forms.office.com/r/ZyLNjuYk3Y. Source Data are provided with this paper.

## Reporting Summary

Further information on research design is available in the Nature Research Reporting Summary linked to this article.

## Code availability

Detect is an open-source anomaly detection framework for neuroimaging data and is available through Github^57^ at github.com/chamberm/Detect under the Apache License. The framework is powered by Streamlit (www.streamlit.io), an open-source app framework for machine learning and data science. The repository is regularly updated via continuous integration to contain example data (tract-profiles), a Wiki section as well as Jupyter Notebooks with the Python code used to generate the figures in this study. A live demo is also available via Streamlit sharing. TractSeg is available at github.com/MIC-DKFZ/TractSeg. MRtrix is available at www.mrtrix.org. SCILPY is available at github.com/scilus/scilpy. FiberNavigator is available at github.com/chamberm/fibernavigator.
