# Supplementary material for: Detecting microstructural deviations in individuals with deep diffusion MRI tractometry
Source: Nat Comput Sci. Author manuscript; Available in PMC 2022 Jul 20. (PMC7613101; doi:10.1038/s43588-021-00126-8)
Supplement: 146348_Sup_Material [file EMS146348-supplement-146348_Sup_Material.pdf]

nature portfolio

https://doi.org/10.1038/s43588-021-00126-8

Supplementary information

Detecting microstructural deviations in individuals with deep diffusion MRI tractometry

In the format provided by the authors and unedited

Supplementary material

CNV and CNP datasets	
a) AF	Arcuate Fasciculus
b) ATR	Anterior Thalamic Radiation
c) CC1	Corpus Callosum (Rostrum)
d) CC2	Corpus Callosum (Genu)
e) CC6	Corpus Callosum (Isthmus)
f) CC7	Corpus Callosum (Splenium)
g) CG	Cingulum
h) CST	Corticospinal Tract
i) IFOF	Inferior Fronto-Occipital Fasciculus
j) ILF	Inferior Longitudinal Fasciculus
k) OR	Optic Radiation
l) SLF-I	Superior Longitudinal Fasciculus I
m) SLF-II	Superior Longitudinal Fasciculus II
n) SLF-III	Superior Longitudinal Fasciculus III
o) UF	Uncinate Fasciculus
**FCD Subject 1 - left temporal lesion**	
AF	Arcuate Fasciculus (left)
IFOF	Inferior Fronto-Occipital Fasciculus (left)
ILF	Inferior Longitudinal Fasciculus (left)
OR	Optic Radiation (left)
UF	Uncinate Fasciculus (left)
**FCD Subject 2 - superior precentral gyrus lesion**	
CC4	Corpus Callosum (primary motor)
CC5	Corpus Callosum (primary sensorimotor)
Cg	Cingulum (right)
CST	Corticospinal Tract (right)
SLF-I	Superior Longitudinal Fasciculus (right)

Supplementary Table 1. List of tracts employed for the three datasets. For the CNV and CNP datasets, bilateral tracts were used.

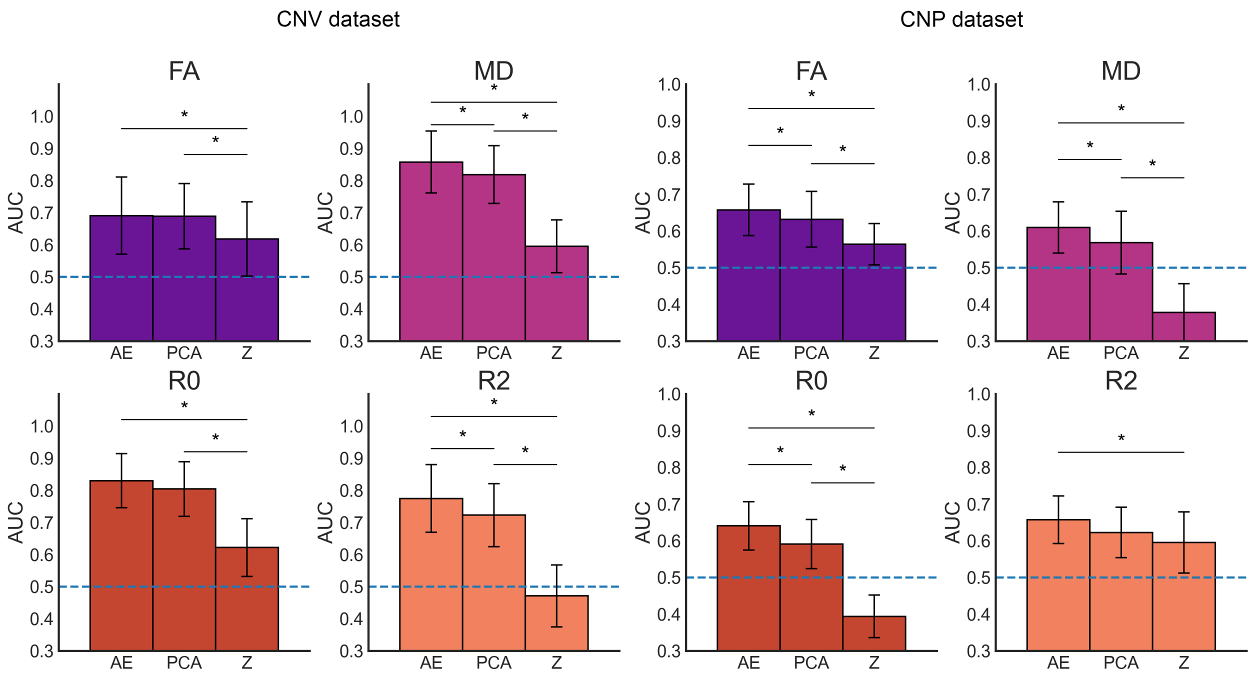

Supplementary Figure 1. Average area under the curve (AUC) scores over 100 iterations for the CNV and CNP datasets. In general, the Autoencoder (AE) approach showed higher AUC scores across the microstructural metrics. *: $p < 8.3e\text{-}4$, Bonferroni corrected with $\alpha = 0.01$, 2-tailed t-tests. FA: Fractional Anisotropy. MD: Mean Diffusivity. RISH0: 0th order rotationally-invariant spherical harmonics feature, RISH2: 2nd order rotationally-invariant spherical harmonics feature. Dashed line: random classifier. Data are presented as mean values +/- standard deviation.

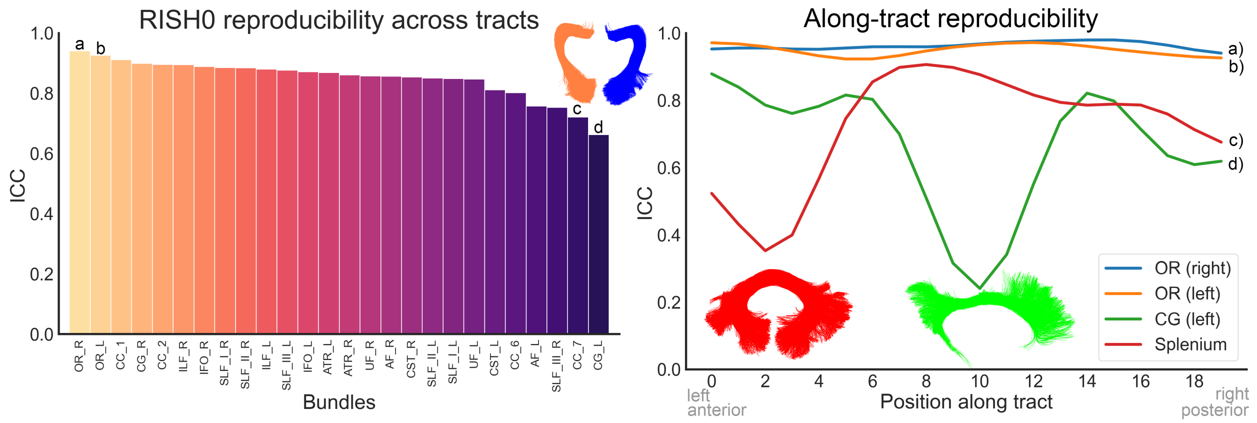

Supplementary Figure 2. Tract-profile repeatability of RISH0. Repeatability was assessed using the intra-class correlation coefficient (ICC, two-way mixed, absolute agreement) computed over 6 subjects (5 time points). Most of the bundles show excellent repeatability (mean ICC: 0.86, mean CoV: 0.03). In particular, the left and right optic radiations ranked amongst the most reproducible bundles, whereas the splenium and left cingulum show the lowest scores. The along-tract ICC profiles revealed anatomical locations where the ICC was lower in those bundles. a) Optic radiation (right), b) Optic radiation (left), c) Splenium (CC_7), d) Cingulum (left).

CNV	FA	MD	RISH0	RISH2
Z-score:	KS=0.3<br>p=0.48<br>Cohen's d=0.35	KS=0.38<br>p=0.21<br>Cohen's d=0.61	KS=0.34<br>p=0.3<br>Cohen's d=0.38	KS=0.16<br>p=0.98<br>Cohen's d=0.1
PCA:	KS=0.47<br>p=0.06<br>Cohen's d=0.28	KS=0.66<br>p=0.001<br>Cohen's d=1.31	KS=0.56<br>p=0.01<br>Cohen's d=1.20	KS=0.49<br>p=0.04<br>Cohen's d=0.81
Autoencoder:	KS=0.45<br>p=0.07<br>Cohen's d=0.18	KS=0.64<br>p=0.002<br>Cohen's d=1.17	KS=0.62<br>p=0.003<br>Cohen's d=1.39	KS=0.55<br>p=0.02<br>Cohen's d=0.9

CNP	FA	MD	RISH0	RISH2
Z-score:	t= 3.41<br>p=0.0008<br>Cohen's d: 0.61	t=-1.79<br>p=0.07<br>Cohen's d: 0.32	t=1.85<br>p=0.07<br>Cohen's d = 0.33	t=3.79<br>p=0.0002<br>Cohen's d: 0.68
PCA:	t=-3.08<br>p=0.002<br>Cohen's d: 0.55	t=-1.51<br>p=0.13<br>Cohen's d: 0.27	t=-1.75<br>p=0.08<br>Cohen's d = 0.32	t=-2.39<br>p=0.02<br>Cohen's d: 0.43
Autoencoder:	t=-3.66<br>p=0.0003<br>Cohen's d: 0.66	t=-2.51<br>p=0.01<br>Cohen's d: 0.45	t=-2.60<br>p=0.01<br>Cohen's d = 0.47	t=-2.80<br>p=0.006<br>Cohen's d: 0.50

Supplementary Table 2. Group statistics and effect sizes (Cohen's d) for the 3 anomaly methods. For the CNV dataset, the Kolmogorov-Smirnov (KS) statistic was employed due to the imbalance between the groups. For the CNP dataset, a 2-tailed t-test assuming equal variances was used.

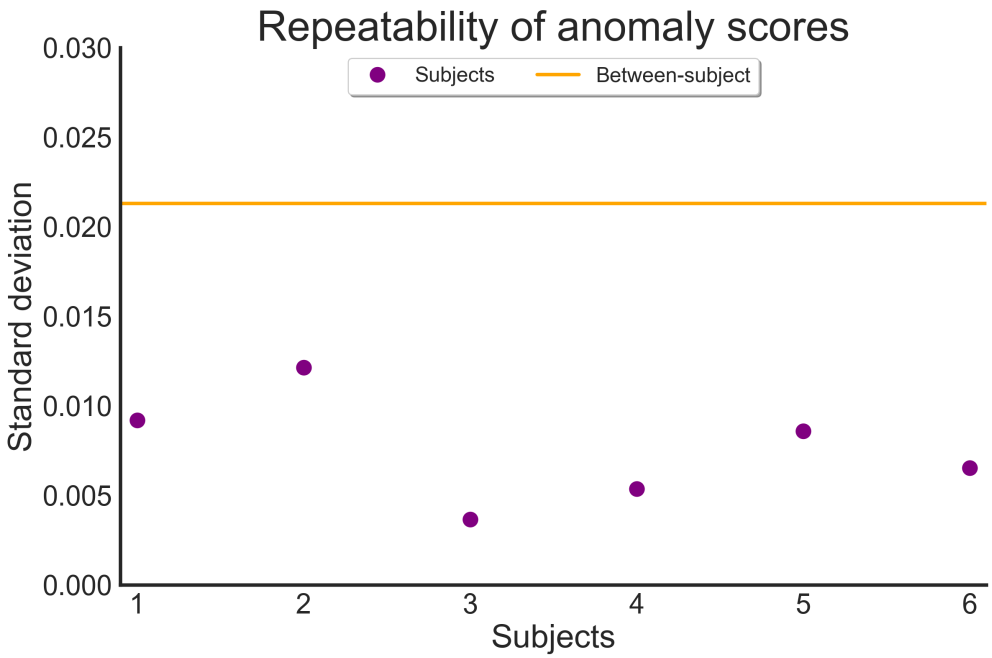

Supplementary Figure 3. Repeatability of anomaly scores derived from the RISH0 features in 6 subjects scanned over 5 sessions. In terms of anomaly scores, the proposed anomaly detection framework shows reconstruction errors that are reproducible across sessions with an ICC of 0.96 (95% CI: 0.88, 0.99), a mean within-subject standard deviation of 0.008 and a CoV of 0.04. The orange horizontal line shows the between-subject standard deviation value (0.02). The fact that the between-subject value is much higher suggests that between-subject differences dominate the dataset and are more prominent than the within-subject differences.

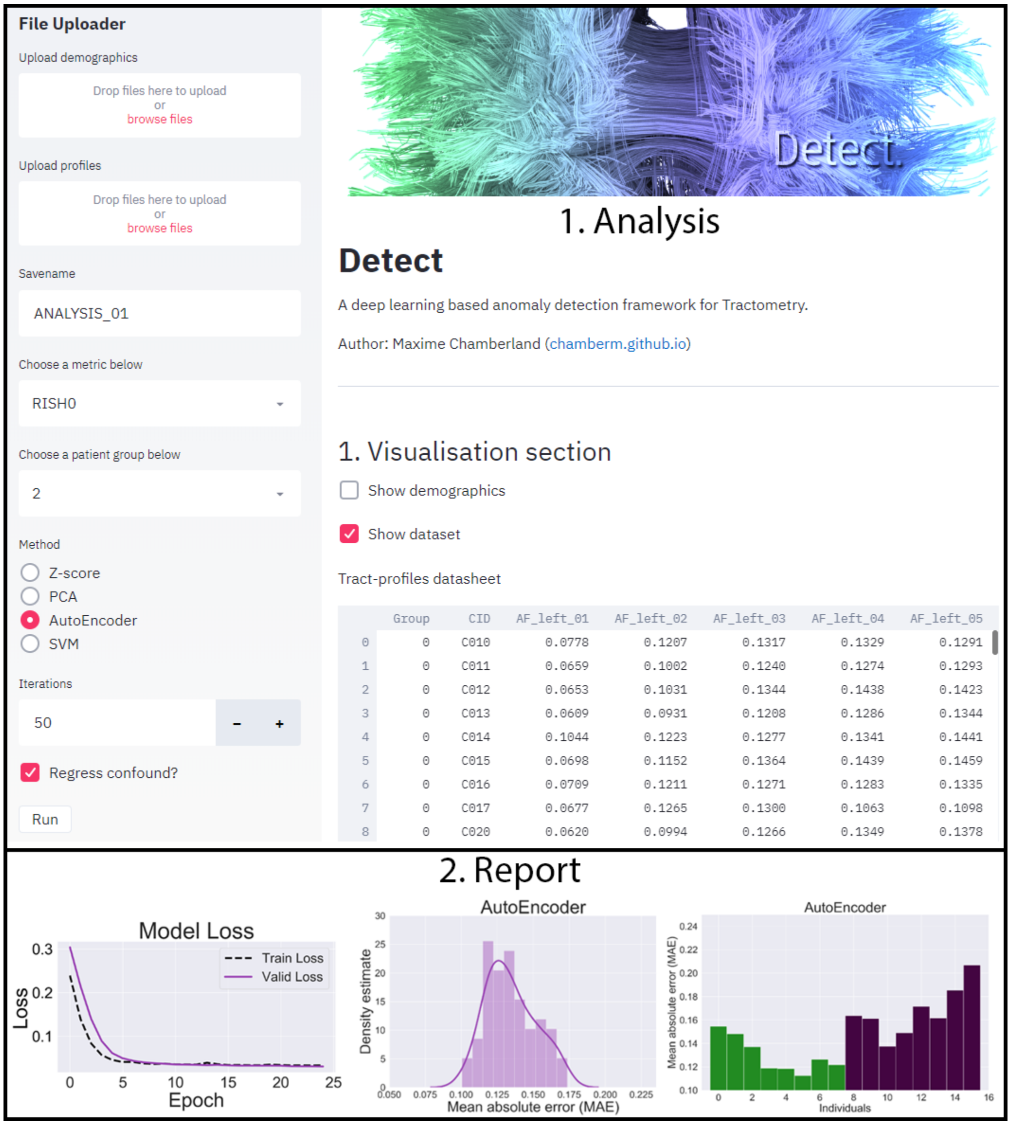

Supplementary Figure 4. Overview of the proposed interactive application in the browser (`github.com/chamberm/detect`). The user has access to various setting on the left panel, including the choice of the diffusion metric, the anomaly method, and the number of iterations. The main display (right) updates in real-time during computation, showing the training loss, the distribution of anomaly scores and the ROC results. At the end, a final report with the computed anomaly scores for each subject is saved for further analysis.
